# A systematic review and meta analysis on burnout in physicians during the COVID-19 pandemic: A hidden healthcare crisis

**DOI:** 10.3389/fpsyt.2022.1071397

**Published:** 2023-01-12

**Authors:** Marie Michele Macaron, Omotayo Ayomide Segun-Omosehin, Reem H. Matar, Azizullah Beran, Hayato Nakanishi, Christian A. Than, Osama A. Abulseoud

**Affiliations:** ^1^School of Medicine, St. George’s University of London, London, United Kingdom; ^2^University of Nicosia Medical School, University of Nicosia, Nicosia, Cyprus; ^3^Division of Gastroenterology and Hepatology, Mayo Clinic, Rochester, MN, United States; ^4^Division of Gastroenterology and Hepatology, Indiana University, Indianapolis, IN, United States; ^5^School of Biomedical Sciences, The University of Queensland, Brisbane, QLD, Australia; ^6^Department of Psychiatry and Psychology, Mayo Clinic Arizona, Phoenix, AZ, United States; ^7^Department of Neuroscience, Mayo Clinic Arizona, Phoenix, AZ, United States

**Keywords:** burnout, healthcare workers, COVID-19, systematic review, meta-analysis, physicians

## Abstract

**Objective:**

This systematic review and meta-analysis aims to explore overall prevalence of burnout among physicians during early and late COVID-19 pandemic and geographical differences in burnout.

**Methods:**

This review was registered prospectively with PROSPERO (CRD42022327959). A comprehensive search of several databases, including Ovid MEDLINE(R) and Epub Ahead of Print, In-Process & Other Non-Indexed Citations and Daily, Ovid Embase, Ovid Cochrane Central Register of Controlled Trials, Ovid Cochrane Database of Systematic Reviews, PsycINFO, and Scopus, spanning from December 2019 to May 2022 was conducted. Eligible studies included physicians or medical professionals including physicians that worked directly or indirectly with COVID-19 patients, whilst reporting burnout outcomes using a validated scale. Literature that did not include physicians or did not occur in a hospital setting were excluded. Literature including medical students were also excluded.

**Results:**

Forty-five observational studies were included, all of which were cross-sectional studies. The pooled estimate of overall prevalence of burnout was 54.60% (95% CI: 46.7, 62.2). Mean emotional exhaustion, depersonalization, and personal accomplishment was found to be 22.06% (95% CI: 18.19, 25.94), 8.72 (95% CI: 6.48, 10.95) and 31.18 (95% CI: 27.33, 35.03) respectively. Frontline workers displayed higher rates of burnout than second-line healthcare workers (HCW) (OR: 1.64, 95% CI: 1.13, 2.37). Studies from the early pandemic period reported burnout prevalence of 60.7% (95% CI: 48.2, 72) compared to a prevalence of 49.3% (95% CI: 37.7, 60.9) from the late pandemic period. Geographically, burnout was highest amongst Middle East and North Africa (MENA) studies (66.6%, 95% CI: 54.7, 78.5), followed by Europe (48.8%, 95% CI: 40.3, 57.3) and then South America (42%, 95% CI: –0.4, 84.4). Lastly, burnout prevalence overall (OR = 0.77, 95% CI: 0.36, 1.67) emotional exhaustion (MD = –0.36, 95% CI: –4.64, 3.91), depersonalization (MD = –0.31, 95% CI: –1.80, 1.18), and personal accomplishment (MD = 0.55, 95% CI: –0.73, 1.83) were found comparable between physicians and nurses.

**Conclusion:**

COVID-19 has had significant consequences on HCW burnout. Further research is needed to examine early signs of burnout and to develop effective coping strategies.

## 1. Introduction

With the Corona virus disease 2019 (COVID-19) pandemic caused by Severe Acute Respiratory Syndrome Corona Virus 2 (SARS-CoV-2) slowly easing its grip on global healthcare concerns and nearing its resolution, attention is now shifting toward the long term imprint this crisis has left worldwide.

One such imprint is job burnout, a psychological syndrome that stems from extended exposure to work-related stressors that occur in workers who interact with other individuals in some capacity, typically that of staff-client. Due to this, job burnout is observed across a variety of occupational sectors, including healthcare, social services, and education and its effects compromise not only the individual but the society as a whole (1).

Measurement of burnout is divided into three distinct dimensions: emotional exhaustion, feelings of cynicism (depersonalization), and a sense of ineffectiveness (lack of personal accomplishment) (2), as described by the most widely used burnout scale, the Maslach Burnout Inventory (MBI) (1). With emotional exhaustion, workers feel emotionally spent and have a sense of apathy regarding their work. Depersonalization is characterized as negative and cynical feelings toward oneself and those one interacts with. These contemptuous feelings are often related to feeling emotionally depleted, hence the correlation between depersonalization and emotional exhaustion. Lack of personal accomplishment describes an overall feeling of unsatisfaction with oneself and their work (1). Other validated scales used for measuring burnout have similar outcomes, including the Professional Quality of Life (ProQOL) scale (3), the Oldenburg Burnout Inventory (OLBI) (4), the Copenhagen Burnout Inventory (CBI) (5), the Stanford Professional Fulfillment Index (PFI) (6, 7).

Individuals with burnout suffer harmful effects of negative emotions, substance abuse and suicidal ideation (8). In physicians, this domino effect may manifest as medical errors, longer recovery times and increased physician turnover due to reduced physician productivity (9). Consequently, these effects pose a greater economic burden to the healthcare system by increasing healthcare costs for replacement of vacancies from resignations (10). Burnout does not only affect physicians, but also carries a toll on all healthcare workers (HCW); frontline workers and nurses in certain countries may be at higher risk of developing burnout. During the pandemic, frontline and second-line HCW were exposed to varying levels of stressors. Frontline workers are defined as those working directly with COVID-19 patients, while second-line workers are defined as HCW with no direct exposure to COVID-19 patients. Though both groups experienced psychological effects due to the nature of their work, frontline workers were more vulnerable due to their proximity with COVID-19 patients (11), (12). Additionally, burnout affects physicians and nurses differently, given that nurses are more intensely exposed to their patients and thus experience more work-related stressors (13). HCW burnout levels have also been shown to be higher in lower income countries than in middle or high income countries (14).

It is not entirely clear whether HCW were more vulnerable to burnout during early pandemic period, when there was shortage of personal protective equipment (PPE), limited knowledge about the illness, and no proper prevention treatment (15, 16) or during the later stages when the prolonged stress accumulated, and the number of infected individuals rose exponentially (17).

The issues of physician burnout have been adequately studied, and have shown a negative effect on depression, stress, mood disorders, suicides, and poor patient quality care (18–20). This remains true for other healthcare professions, as high nurse burnout has been linked with increased turnover, leading to nurse shortage and poorer patient care outcomes (21). Although these phenomena have been studied, measures have not been taken to alleviate this issue. Understanding the subtle differences in burnout domains between different groups and at different geographical locations could provide valuable guidance in developing effective intervention strategies.

As frontline HCW continue to respond to the COVID-19 outbreak, it is of utmost importance that we invest immediately in the psychological wellness of HCW. Burnout in medical professionals has generally been overlooked and the novelty of the COVID-19 outbreak presents a gap in understanding burnout prevalence trends in healthcare in such unforeseen situations. It is in the best interest of public health to start acting on this issue now before the burnout-related effects progress any further. Therefore, the aim of this meta-analysis is to explore burnout prevalence in HCW, focusing on physicians, during the COVID-19 pandemic as well as differences in burnout prevalence according to region, COVID-19 timelines, and healthcare profession (whether nurse or physician) to better understand this hidden healthcare crisis.

## 2. Methods

### 2.1. Search strategy and data sources

The review followed the Preferred Reporting Items for Systematic Reviews and Meta-analyses (PRISMA) guidelines (22). A comprehensive search of several databases from December 2019 to May 2022 was conducted for pertinent English language publications. The databases included Ovid MEDLINE(R) and Epub Ahead of Print, In-Process & Other Non-Indexed Citations and Daily, Ovid Embase, Ovid Cochrane Central Register of Controlled Trials, Ovid Cochrane Database of Systematic Reviews, PsycINFO, and Scopus. The search strategy was designed and conducted by an experienced librarian. Controlled vocabulary with keywords was used to search for studies describing COVID-19 and physician burnout. Supplementary Item 1 outlines the search strategy listing all the search terms used and how they are combined. This review was registered prospectively with PROSPERO (CRD42022327959).

### 2.2. Eligibility criteria and quality assessment

Eligible studies were observational studies that met all the following inclusion criteria: (1) Studies of physicians or medical healthcare professionals including physicians who were frontline workers or a mix of frontline and second-line workers during the COVID-19 pandemic in a hospital setting; (2) Reported burnout outcomes using a validated questionnaire. With regards to study designs, original, observational studies excluding case reports, case series, abstracts, conference abstracts, and articles that were not reported in English. The study also excluded medical school students and studies that did not delineate results between medical and non-medical HCW (e.g., administration, security staff). There was no comparison group in our study. The quality of each study was independently evaluated by two authors (MM and OS-O) using the Newcastle Ottawa Assessment Scale. The scale assesses sample Selection (representativeness of the target population, sample size, comparability between respondents and non-respondents, and outcome ascertainment), Comparability (comparability between subjects in different outcome groups), and Outcomes (method of measurement of outcome and statistical test used). A maximum of one star can be given to a study in each of the categories under Selection and Outcome, and a maximum of two stars can be given for Comparability (23). The difference in the determination of quality was resolved by discussion with a third author until a consensus was reached (RM). Results of the quality assessment of all included studies are shown in Supplementary Item 2.

### 2.3. Questionnaires

#### 2.3.1. Maslach burnout inventory

The MBI scale measures burnout with 22 items measuring three dimensions: nine items measuring emotional exhaustion, five measuring depersonalization, and eight measuring personal accomplishment. Traditionally, high scores on both emotional exhaustion and depersonalization and low scores on personal accomplishment were associated with increased burnout risk (24). Each item is measured using a five- or seven-point Likert scale depending on the study. Some of the included studies used a modified MBI were only one (emotional exhaustion) (25) or two (emotional exhaustion and depersonalization) (26, 27) of the MBI subscales were used to evaluate burnout in their population.

#### 2.3.2. Professional quality of life

The ProQOL scale measures the effects of traumatic stress experience on work burnout, compassion satisfaction, and compassion fatigue. Each subscale has ten items and cut-offs depend on the type of Likert scale used. Traditionally, a five-point Likert scale is employed, and for each subscale, moderate levels of burnout are indicated by scores of 23–41, whereas scores over 41 indicate high levels (28).

#### 2.3.3. Oldenburg burnout inventory

The OLBI consists of two subscales, exhaustion, and disengagement, measured by eight positively and negatively framed items each. It is used as an alternative to the MBI so that the subscales of depersonalization and personal accomplishment are seen as consequences of stress and coping (29). The OLBI scale generally uses a four-point Likert scale going from one (strongly agree) to four (strongly disagree). Higher scores are associated with worse burnout symptoms and in most studies, burnout is defined as positive when the total score is greater or equal to 21 (30).

#### 2.3.4. Copenhagen burnout inventory

The CBI consists of nineteen items and three different subscales which apply to a greater range of occupational sectors; personal related burnout (six items), work-related burnout (seven items), and client-related burnout (six items) (31). The work-related burnout sector uses a 5-point Likert scale, with higher scores indicating higher levels of burnout (32).

#### 2.3.5. Stanford professional fulfillment index

The Stanford PFI was developed to evaluate both burnout and physician professional well-being at work, with the two domains of burnout represented as interpersonal disengagement and work-exhaustion (33). Hence, the PFI is a sixteen-items scale with four items assessing work-exhaustion, six items evaluating professional fulfillment, and six items assessing interpersonal disengagement. Possible burnout scores range from zero to forty for burnout and zero to 24 for professional fulfillment (34).

#### 2.3.6. Mini-Z survey

The Mini-Z burnout survey is a twelve-item measurement scale, with items one through eleven being five- point Likert scale assessing job satisfaction, stress, burnout, satisfactory control of working conditions, satisfactory time available for documentation of cases, chaos, professional value alignment with those of the department heads, good teamwork, time spent on electronic medical record at home, and gender or racial discrimination. Item 12 is the following open question: “Tell us more about your stresses and what we can do to minimize them” (7).

### 2.4. COVID timelines

In this paper, early pandemic period referred to the onset of the pandemic until August 2020, while late pandemic period referred to September 2020 onward, based off of general trends of the first (February 2020 to August 2020), second (September 2020 to Mid-February 2021), and third (Mid-February 2021 to June 2021) COVID-19 waves (35).

### 2.5. Geographical locations

We categorized studies into six regions, three with high income countries: Europe (Cyprus, France, Italy, Romania, Russia, and Spain), North America (Canada and the USA), and Australia, and three with middle/low-income countries: Asia (China, India, Malaysia, Pakistan, Singapore, South Korea, Taiwan, Turkey), Middle East and North Africa (MENA) (Egypt, Iran, Jordan, Qatar, Saudi Arabia), and South America (Argentina and Brazil).

### 2.6. Statistical analysis

The pooled means and proportions of our data were analyzed using a random, inverse variance method for continuous data and the Mantel-Haenszel method for dichotomous data. The heterogeneity of effect size estimates across the studies was quantified using the Q statistic and the *I*^2^ index (*P* < 0.10 was considered significant). A value of *I*^2^ of 0–25% indicates minimal heterogeneity, 26–50% moderate heterogeneity, and 51–100% substantial heterogeneity (36). Data analysis was performed using Open Meta analyst software (CEBM, Brown University, Providence, Rhode Island, USA) and RevMan software version 5.4 (Review Manager (RevMan) [Computer program]. The Cochrane Collaboration, 2020, Copenhagen, Denmark). If mean and standard deviation (SD) were unavailable, median was converted to mean using the formulas from the Cochrane Handbook for Systematic Reviews of Interventions (37). Authors were contacted three times to obtain additional information such as the exact Likert scale used in the MBI scale, clarification of setting (hospital or outpatient), and whether the population included only medical healthcare workers or non-medical staff as well. Publication bias was assessed using a funnel plot (38).

### 2.7. Data extraction

Following a thorough reading of the articles, the necessary information was retrieved using the summary and collection form. The title, responsible author, the sample size of the study, country and time of the study, study design, study participants based on their patient-facing roles (doctor, nurse, and other clinical), exposure of the participants to COVID-19 patients in the workplace, diagnostic instrument, and findings were all provided on this form. For each of the selected articles, summary forms were filled.

Necessary information was extracted from the articles and rechecked by two authors (MM and OS-O). The extraction included general information about the studies (title, author, study year and timeline of data collection, country, study design, and study setting), population characteristics (sample size, population gender, mean age, and professional role i.e., physician, nurse, or allied healthcare worker) and outcomes which included reported scores on the respective burnout scales used in each study.

## 3. Results

### 3.1. Identification and selection of studies

A literature search of several databases following PRISMA guidelines yielded 2,361 records, from which 2,147 records were eliminated by title and abstract screening. The remaining 163 records were then assessed for full text screening and 45 records were finally included in the study. Out of these 45 included studies, twenty were utilized for quantitative analysis. The PRISMA flow diagram is shown in Supplementary Item 3.

### 3.2. Study characteristics

All the 45 observational studies included were cross-sectional. A total of 29,785 medical healthcare professionals were included in the study population. Mean age range of workers was 27–47 years. A total of 27 (8, 26, 27, 39–59) studies included both frontline and second-line workers in their studies, while twelve studies (24, 25, 28, 60–68) had only frontline workers and six (32, 69–73) did not delineate between frontline and second-line workers. In terms of regions, fifteen (33.3%) of studies (28, 41, 45, 47, 51, 55, 56, 58, 59, 63, 67, 68, 71, 72, 74) took place in Asia. Europe (8, 26, 42, 48, 53, 57, 60, 62, 66, 69, 73) was represented by 11 (24.4%) studies while the Middle East and North Africa (MENA) (25, 32, 39, 44, 46, 49, 50, 64, 65, 70) were represented by ten (22.2%) studies each. South America (24, 40, 52, 54, 61, 75), North America (27, 76), and Australia (43) were represented by six (13.3%), two (4.4%), and one (2.2%) study respectively. Burnout results between nurses and physicians were compared in fourteen studies (32, 42, 43, 53, 55, 56, 62, 63, 67–71, 74). Twenty-eight studies (8, 25, 27, 28, 32, 39, 40, 42–44, 47, 49, 50, 52, 53, 55–59, 62, 63, 65, 67, 68, 70, 73, 75) conducted data collection during the early stages of the COVID-19 pandemic and fifteen (24, 26, 41, 44–46, 48, 51, 54, 60, 62, 66, 69, 71, 74, 76) studies were conducted during the later stages. Two studies (26, 61) did not specify the time of data collection. Burnout was measured using a variety of questionnaires. Thirty studies (8, 24–27, 39–42, 44, 46, 48, 49, 53, 54, 57–59, 61–64, 66, 68, 70–72, 74, 76) used the MBI questionnaire or some variation of it. All other information regarding sample size, timeline of data collection, and age distribution are included in Table 1.

**TABLE 1:** Study and baseline characteristics.

Citation	Timeline	Year of publication	Country	Burnout tool	Frontline (% of frontline)	Second-line (% of second-line)	Setting	Sample size (n)	Study participants role	Age (SD)*	Married/ Partner	Female (n)
Akova et al. (74)	1 Sep 2021–1 Oct 2021	2022	Turkey	MBI (5-point Likert)	852 (84.4)	163 (15.6)	NR	1,015	Nurse (252) Physicians (569) Medical Assistants (388)	NR	738	482
Alsulimani et al. (32)	Jun 2020-Aug 2020	2021	Saudi Arabia	CBI	NR	NR	ICU (55) ER (102)	640	Nurse (301) Physicians (71) Residents (226) Medical assistants (42)	NR	NR	NR
Alwashmi and Alkhamees (39)	27 May 2020–8 Aug 2020	2021	Saudi Arabia	MBI (7-point Likert)	26 (25.7)	75 (74.3)	NR	101	Physicians (43) Residents (58)	NR	65	56
Appiani et al. (40).	6 May 2020–8 Aug 2020	2021	Argentina	MBI (unspecified Likert range)	138 (45.7)	164 (54.3)	ER (10)	302	Physicians (152) Residents (103) Department Head (47)	NR	NR	155
Asghar et al. (41)	17 Nov 2020–1 Jan 2021	2021	Pakistan	MBI (7-point Likert)	52 (59.8)	35 (40.2)	NR	87	Physicians (18) Residents (69)	30.87 (7.34)	45	47
Azoulay et al. (69)	30 Oct 2020–1 Dec 2020	2021	France	MBI (7-point Likert)	NR	NR	ICU (845)	845	Nurse (412) Physicians (175) Residents (97)	33	NR	571
Babamiri et al. (70)	16 May 2020–22 May 2020	2022	Iran	MBI (7-point Liker	NR	NR	NR	242	Nurse (86) Physicians (76) Medical Assistant (80)	NR	NR	NR
Di Mattei et al. (42)	9 May 2020–13 Jul 2020	2021	Italy	MBI (7-point Likert)	331 (35.9)	590 (64.1)	NR	921	Nurse (362) Physicians (99) Medical Assistants (105)	NR	NR	NR
Dobson et al. (43)	16 Apr 2020–13 May 2020	2021	Australia	SPFI	120 (41.4)	170 (58.6)	NR	290	Nurse (86) Physicians (99) Medical Assistants (105)	NR	NR	223
Enea et al. (60)	19 Oct 2020–28 Oct 2020	2021	Romania	CBI	110 (100)	NR	ICU (110)	110	Nurse (39) Physicians (76) Medical assistants (2)	43.64 (12.09)	23	88
Etesam et al. (44)	First half of the year 2020	2021	Iran	MBI (7-point Likert)	100 (82)	22 (18)	COVID wards (149)	122	NR	NR	NR	NR
Fumis et al. (24).	10 Dec 2020–23 Dec 2020	2021	Brazil	MBI (5-point Likert)	51 (100)	NR	ICU (51)	51	Physicians (51)	37 (7.41)	39	20
Gupta et al. (45)	1 Aug 2020–15 Dec 2020	2021	India	Mini-Z	664 (41.1)	951 (58.9)	NR	1615	Nurse (509) Physicians (263) Medical assistants (223)	37.74 (10.73)	1211	631
Haji Seyed Javadi et al. (46)	10 Dec 2020–16 Apr 2021	2021	Iran	MBI (7-point Likert)	187 (48.7)	197 (51.3)	NR	384	Nurse (293) Physicians (39) Medical Assistants (52)	40.01 (11.90)	299	NR
Ibar C et al. (61)	Not specified	2021	Argentina	MBI (7-point Likert)	133 (100)	NR	NR	133	Nurse (925) Physicians (34) Residents (35) Medical Assistants (39)	NR	NR	NR
Ismail et al. (25)	Apr 2020-Aug 2020	2021	Egypt	MBI (7-point Likert 1–7)	150 (100)	NR	NR	150	Physicians (150)	28.6 (10.8)	60	20
Jiang et al. (28)	Mar 2020–Apr 2020	2021	China	ProQOL	219 (100)	NR	ICU (73)	219	Nurse (219)	31.17 (4.99)	145	176
Kanneganti et al. (47)	29 May 2020–13 Jul 2020	2022	Singapore, Malaysia, India, Malaysia	OLBI	471 (17)	2, 301 (83)	NR	2,772	Nurse (1,470) Physician (878) Medical Assistants (424)	33.3 (8.2)	NR	NR
Kapetanos et al. (62)	May 2020–Jun 2020	2021	Cyprus	MBI (7-point Likert)	351 (100)	NR	NR	351	Nurse (277) Physicians (49) Medical assistants (25)	NR	NR	248
Karacan et al. (63)	1 May 2020–30 Jun 2020	2021	Turkey	MBI (unspecified 7-point Likert)	497 (100)	NR	ICU (86) ER (89)	497	Nurse (97) Physicians (150) Residents (76) Medical Assistants (174)	NR	NR	NR
Kashtanov et al. (48)	Jan 2021–Jul 2021	2022	Russia	MBI (7-point Likert)	956 (75.9)	303 (24.1)	ICU (889)	1,259	Nurse (492) Physicians (767)	36.28 (12.03)	NR	575
Khan et al. (76)	Aug 2020–Oct 2020	2021	Canada	MBI (7-point Likert)	49 (19.7)	200 (80.3)	ICU (16)	249	Physicians (249)	NR	NR	122
KhoodoruthMAS et al. (50)	17 May 2020–16 Jun 2020	2021	Qatar	ProQOL	80 (63)	47 (37)	NR	127	Residents (127)	NR	62	48
Kim et al. (51)	Apr 2021–May 2021	2021	South Korea	ProQOL	178 (72.1)	69 (27.9)	ER (247)	247	ER Physician (137) Residents (110)	NR	NR	63
Mendonça et al. (75)	Apr 2020–Apr 2020	2021	Brazil	OLBI	973 (69.9)	419 (30.1)	NR	1,392	Residents (1,392)	27.9 (3.0)	NR	1,010
Mosolova et al. (26)	Not specified	2020	Russia	MBI-EE and DP	455 (41.2)	650 (58.8)	NR	1,105	Nurses (164) Physicians (941)	34 (12.59)	NR	742
Mousavi-Asl et al. (64)	Not specified	2020	Iran	MBI (7-point Likert)	87 (100)	NR	COVID-19 special wards (87)	87	NR	30.86 (0.63)	49	22
Mutleq et al. (65)	1 May 2020–20 May 2020	2020	Jordan	OLBI	124 (100)	NR	NR	124	Nurse (39) Physicians (39)	NR	29	95
Naldi et al. (53)	27 Apr 2020–May 2020	2021	Italy	MBI (7-point Likert 1–7)	563 (70.6)	234 (29.4)	NR	797	Nurse (469) Physicians (328)	NR	564	599
Queiroz de Paiva Faria AR et al. (54)	Nov 2020–Nov 2020	2021	Brazil	MBI (5-point Likert)	82 (65.1)	44 (34.9)	COVID-19 ward (49) ICU (33)	126	Physicians (126)	NR	88	81
Ruiz-Fernanadez MD et al.(73)	Mar 2020-April 2020	2020	Spain	ProQOL	NR	NR	ICU (65) Emergency department (76) Regular hospital care (140) COVID-19 Unit (30)	311	NR	NR	NR	NR
Sarikhani et al. (49)	Mar 2020–Jan 2021	2021	Iran	MBI (7-point Likert)	326 (75.5)	106 (24.5)	COVID-19 (283)	432	Physicians (212) Residents (220)	31.32 (8.89)	162	222
Shiu et al. (55)	12 Mar 2020–29 Mar 2020	2021	Taiwan	Single Item Study	560 (39.4)	861 (60.6)	NR	1,421	Nurse (1,064) Physicians (357)	36.64 (8.13)	NR	1,159
Singh et al. (27)	21 Jun 2020–21 Aug 2020	2022	USA	MBI (modified 7-point)	494 (79.7)	126 (20.4)	NR	620	Nurse (524) Residents (96)	46.51 (13.3)	464	300
Steil et al. (52)	Apr 2020	2022	Brazil	OLBI	1,926 (62.7)	1,145 (37.3)	NR	3,071	Residents (3,071)	28 (3.2)	NR	2,311
Stocchetti et al. (66)	11 Jan 2021–28 Jan 2021	2021	Italy	MBI (7-point Likert)	136 (100)	NR	ICU (136)	136	Nurse (84) Physicians (52)	39.1 (11.25)	86	79
Teo et al. (56)	12 Mar 2020–21 Apr 2020	2021	Singapore	One item Physician Work Life Scale	781 (76.1)	245 (23.9)	N	1,026	Nurse (822) Physicians (204)	35.1 (10.27)	NR	799
Torrente et al. (57)	21 Apr 2020–3 May 2020	2021	Spain	MBI- (5-point Likert)	377 (58.6)	266 (41.4)	NR	643	Nurse (172) Physicians (408) Medical assistant (63)	NR	491	472
Treluyer and Tourneaux (8)	1st week May 2020–11 May 2020	2021	France	MBI (8-point Likert)	136 (40)	204 (60)	NR	340	Physicians (340)	27 (2.22)	242	285
Tuna and Ozdin S (58)	23 Apr 2020–27 Apr 2020	2021	Turkey	MBI (5-point Likert)	188 (46.3)	218 (53.7)	NR	406	Physicians (406)	42.9 (10.1)	297	189
Turan et al. (60)	May 2020–Jul 2020	2022	Turkey	MBI (5-point Likert)	33 (82.5)	7 (17.5)	ER (5)	40	ER physicians (5) Physicians (40)	40 (6.35)	38	13
Yilmaz et al. (63)	1 Oct 2020–31 Oct 2020	2021	Turkey	MBI (5-point Likert)	NR	NR	NR	479	Nurse (192) Physicians (287)	NR	381	NR
Zakaria et al. (67)	8 May 2020–15 May 2020	2021	Malaysia	The questionnaire form was adopted from Michelle Post, Public Welfare, Vol. 39, No. 1, 1981, American Public Welfare Association. – Burnout N value	216 (100)	NR	ER (216)	216	ER physicians (4) Nurse (142) Physicians (37) Medical Assistants (37)	30	NR	148
Zhang et al. (68)	18 Feb 2020–4 Mar 2020	2021	China	MBI (7-point Likert)	1,081 (100)	NR	NR	1,081	Nurses (642) Physicians (314) Medical assistants (125)	NR	NR	NR
Zhou et al. (72)	Oct 2020–Oct 2020	2022	China	MBI (7-point Likert)	NR	NR	NR	3,203	Nurse (1,794) Physicians (829) Medical assistants (580)	NR	NR	NR

CBI, Copenhagen burnout inventory; ER, emergency room; Hrs/wk, hours worked per week; ICU, intensive care unit; MBI, Maslach burnout inventory; Mini-Z, zero burnout program; NR, not reported; OLBI, Oldenburg burnout inventory; ProQOL, professional quality of Life; SD, standard deviation; SPFI, Stanford professional fulfillment index; Yrs, years.

### 3.3. Pooled estimated prevalence of burnout and its main domains

**FIGURE 1:**
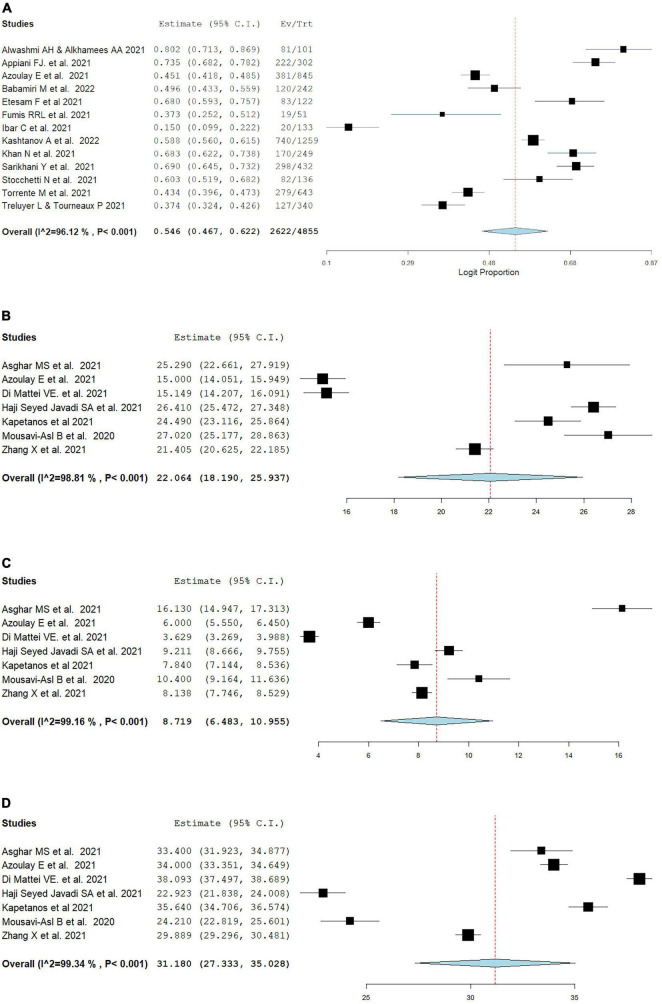
Pooled estimate of overall burnout in healthcare workers according to the Maslach burnout inventory by **(A)** overall prevalence, **(B)** emotional exhaustion, **(C)** depersonalization, **(D)** personal accomplishment.

As shown in Figure 1, overall burnout was evaluated among thirteen studies (8, 24, 39, 40, 44, 48, 49, 57, 61, 66, 69, 70, 76) with MBI or a modified MBI, and the prevalence was 54.6% (95% CI: 46.7, 62.2, *I*^2^ = 96.12%). Mean emotional exhaustion, depersonalization, and personal accomplishment were found to be 22.06 (95% CI: 18.19, 25.94, *I*^2^ = 98.81%), 8.72 (95% CI: 6.48, 10.96, *I*^2^ = 99.16%), and 31.18 (95% CI: 27.33, 35.03, *I*^2^ = 99.34%) respectively.

### 3.4. Burnout in frontline vs second-line HCW

**FIGURE 2:**
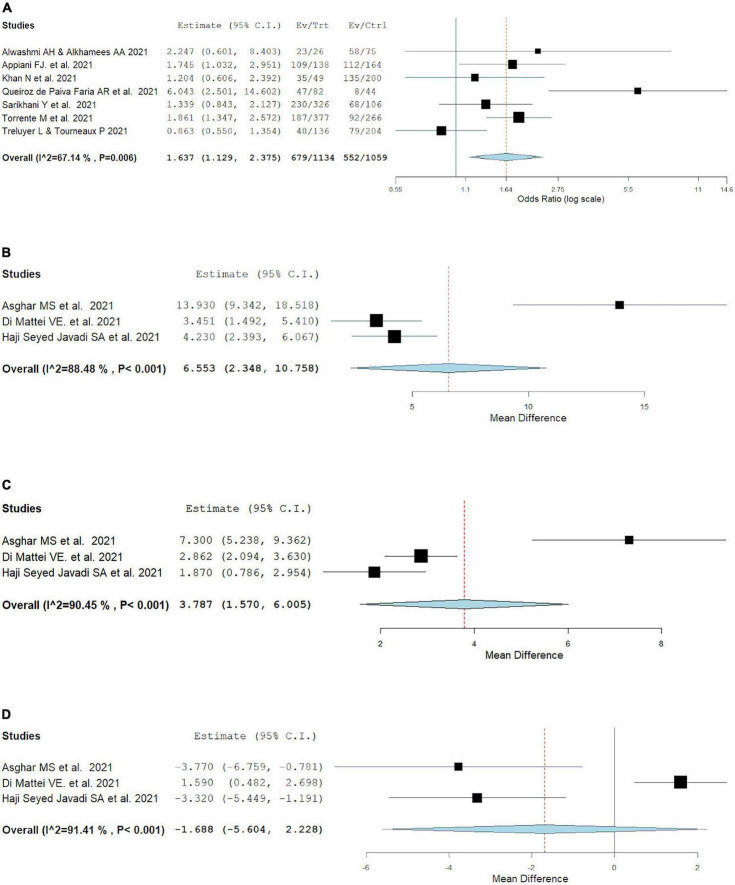
Pooled estimate of burnout in frontline vs second-line healthcare workers according to Maslach burnout inventrory by **(A)** overall prevalence, **(B)** emotional exhaustion prevalence, **(C)** depersonalization prevalence, **(D)** personal accomplishment prevalence.

Overall burnout was evaluated with the MBI scale, and the frontline workers had a higher rate of burnout compared to second-line HCW (OR: 1.64, 95% CI: 1.13, 2.38, *I*^2^ = 67.14%), as can be seen in Figure 2. Mean emotional exhaustion and depersonalization were found to be higher in frontline than in second-line HCW (MD = 6.55, 95% CI: 2.35, 10.76, *I*^2^ = 88.48%) and (MD = 3.79, 95% CI: 1.57, 6.01, *I*^2^ = 90.45%) respectively. Mean personal accomplishment had comparable levels in frontline workers versus second-line workers (MD = –1.69, 95% CI: –5.60, 2.23, *I*^2^ = 91.41%).

### 3.5. Prevalence of burnout during early vs late pandemic

**FIGURE 3:**
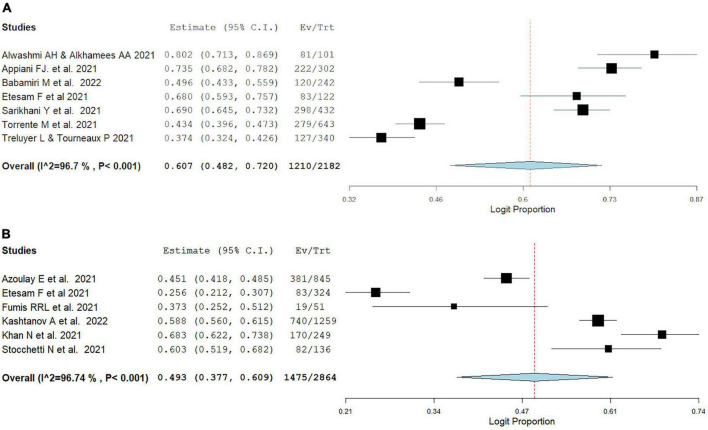
Pooled estimate of burnout prevalence according to the Maslach burnout inventory in **(A)** early pandemic period, **(B)** late pandemic period.

Overall burnout prevalence in the early wave of the pandemic was found to be 60.7% (95% CI: 48.2, 72.0%, *I*^2^ = 96.7%) of the population, as evident in Figure 3. Overall burnout in the later pandemic period was shown to be prevalent in 49.3% (95% CI: 37.7, 60.9%, *I*^2^ = 96.74%) of the population.

### 3.6. Prevalence of burnout in different geographical locations

**FIGURE 4:**
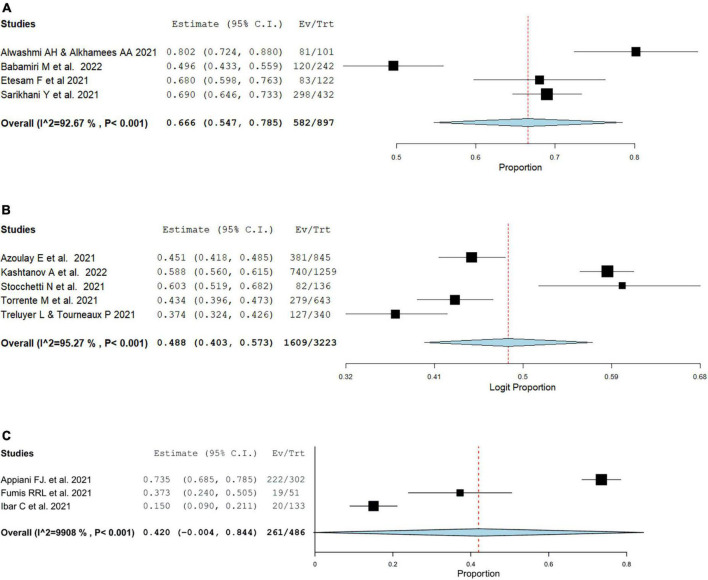
Pooled estimate of burnout prevalence according to Maslach burnout inventory in **(A)** MENA, **(B)** Europe, **(C)** South America. Asian studies forest plots could not be pooled due to burnout scale discrepancy.

As can be seen in Figure 4, burnout prevalence among HCW was highest amongst MENA studies (66.6%, 95% CI: 54.7, 78.5%, *I*^2^ = 92.67%), second highest prevalence was observed in Europe (48.8%, 95% CI: 40.3, 57.3%, *I*^2^ = 95.27%) reporting burnout, and finally the lowest burnout prevalence was found in South America (42%, 95% CI: –0.4, 84.4%, I^2^ = 99.08%). We could not estimate the pooled prevalence in Asian studies because they used different scales to measure burnout.

### 3.7. Prevalence of burnout among nurses vs physicians

**FIGURE 5:**
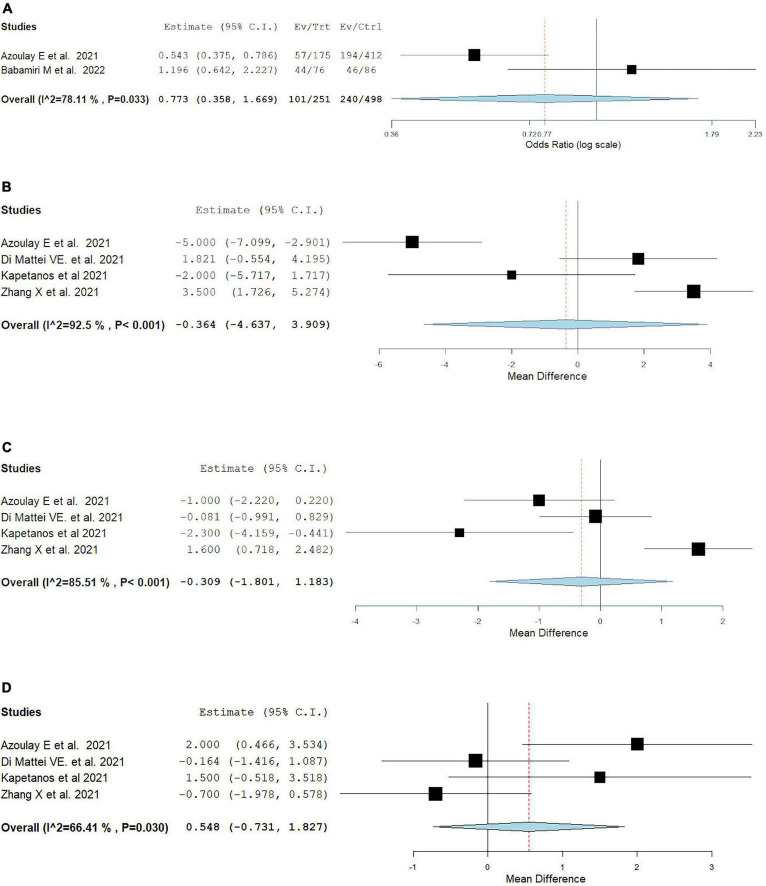
Pooled estimate of burnout prevalence in nurses vs physician according to Maslach burnout inventory by **(A)** overall prevalence, **(B)** emotional exhaustion prevalence, **(C)** depersonalization prevalence, **(D)** personal accomplishment prevalence.

Fourteen studies compared burnout outcomes between physicians and nurses. Among fourteen studies, four studies (42, 62, 68, 69) reported emotional exhaustion, depersonalization, and personal accomplishment prevalence, while two studies (69, 70) reported overall MBI burnout. In comparison to nurses, physicians had comparable rates of overall burnout rate (OR = 0.77, 95% CI: 0.36, 1.67, *I*^2^ = 78.11%). Emotional exhaustion, depersonalization, and personal accomplishment mean result were also comparable between physicians and nurses (MD = –0.36, 95% CI: –4.64, 3.91, *I*^2^ = 92.5%) (MD = –0.31, 95% CI: –1.80, 1.18, *I*^2^ = 85.51%) (MD = 0.55, 95% CI: –0.73, 1.83, *I*^2^ = 66.41%), as showcased in Figure 5.

## 4. Discussion

The COVID-19 pandemic has proven to be an enormous burden on healthcare systems across the globe, placing considerable strain on the psychological well-being of all healthcare workers (HCW) involved. The results of this meta-analysis demonstrate that more than half of HCW experienced burnout at some point during the pandemic. As expected, frontline, compared to second-line HCWs, were found to have higher rates of burnout. In addition, burnout prevalence was shown to be higher during the early pandemic as compared to late and specifically in MENA countries. Finally, physicians and nurses were found to be comparable in overall burnout and all its domains. This meta-analysis specifically examines the prevalence of specific burnout domains which could be utilized as potential targets for therapeutic intervention. To our knowledge, no previous meta-analysis has investigated burnout based on frontline versus second-line HCW, nurses versus physician, early versus late pandemic waves, as well as between regions. Additionally, previous reviews included medical and non-medical healthcare workers, in and out of hospital setting, while this review focused specifically on medical healthcare staff within a hospital setting. The cut-off scores for low, moderate, and high levels of the MBI subdomains used in this review may be found in Supplementary Item 4.

Lack of personal accomplishment, defined as having a negative outlook on the worth of one’s work (1), was found to be the most affected domain during the COVID-19 pandemic out of the three subscales of the MBI. Indeed, the mean score for personal accomplishment referred to a low score as per MBI subscale cut-off ranges, while mean scores for emotional exhaustion and depersonalization were found to indicate moderate levels (77). Increased stress levels are linked to reduced personal accomplishment and feelings of low self-esteem (78). This could be due to increased workload, inadequate protective personal equipment, increased risk of infection, emotional strain of caring for patients suffering alone in confinement (79), and difficult decision making due to scarce resource allocation (80). All these factors were undoubtedly experienced by HCWs during the pandemic, which could lead to a reduced level of effectiveness of care that may affect one’s outlook on their accomplishments as healthcare providers, especially in the case of frontline workers (54).

Frontline workers were at higher risk of experiencing burnout compared to their second-line worker colleagues. They also experienced higher mean scores for emotional exhaustion and depersonalization. Aside from fear of getting infected or spreading the infection to their loved ones, frontline HCW became the target of stigmatization in their communities, with people viewing this group as a possible cause of virus transmission (81). Frontline workers also experienced increased workload coupled with the unique demands of a novel pandemic and reported elevated levels of psychological outcomes such as depression, post-traumatic disorder, and anxiety (12). This in turn provides feasible rationale for the disparity in reported burnout levels seen between frontline and second-line HCWs. However, shortage of HCWs also led to increased workload of second-line workers, leading to higher prevalence of poor sleep quality and anxiety seen in this cohort (82). Further insight into prolonged high workload effect on the mental health and well-being of HCWs could be elucidated by comparing burnout prevalence in early and late pandemic periods.

The early pandemic period was found to be associated with higher prevalence of burnout compared to late pandemic period. However, a study by Melnikow et al. explored burnout prevalence in the first and second waves of the pandemic and found an increase in overall burnout prevalence in the second wave compared to the first wave (83). They also reported that the increased burnout prevalence applied to all frontline specialties except emergency medicine, who displayed reduced burnout results in the second wave compared to the first (Professional Fulfillment Index Burnout Composite Scale score difference: –0.09, 95% CI: –0.53, 0.34), though these differences were not significant. As the pandemic ensued, increased workload, cases, and strain on resources could explain the increase in burnout across the waves in non-emergency specialties as reported by Melnikow et al. Yet, despite this strain, the exercise of various personal resilience and institutional strategies to manage burnout would explain the observed prevalence rates of burnout being lower in the second wave when compared to the first. Also, COVID-19 knowledge and preparedness increased over time thus decreasing COVID-19 fear, which has been shown to correlate with higher levels of burnout among healthcare professionals (84). On the other hand, the differences in prevalence could be attributed to several confounding factors given that the early and late pandemic studies were conducted with different populations, with different sample sizes, and in different countries and regions.

Out of the three regions studied, HCW in MENA displayed a higher prevalence of burnout compared to Europe and South America (66.6, 48.8, and 42%). It is important to note that a contributor to this observation is the number of included studies. South America had the least amount of included studies for the subgroup analysis and an overall total of six studies (24, 40, 52, 54, 61, 75) whereas MENA (25, 32, 39, 44, 46, 49, 50, 64, 65, 70) and Europe (8, 26, 42, 48, 53, 57, 60, 62, 66, 69) both had ten included studies each. We could not estimate pooled prevalence from Asian studies due to wide variability in rating scales. MENA countries report a scarcer number of resources aimed at alleviating burnout compared to European countries (85). Other differences in prevalence can be attributed to cultural differences as well as variation in healthcare systems, as cultural differences may explain the roles physicians play in addition to patient’s attitudes toward their healthcare providers (86). The cultural context of healthcare systems is also thought to play a role in physician versus nurses’ social acknowledgment, affecting their mental health status (87).

Physicians were found to have a comparable risk of burnout with nurses, as well as comparable mean scores for emotional exhaustion, depersonalization, and personal accomplishment. These results are consistent with the findings of another systematic review by Kunz M., Strasser M., and Hasan A 2019 (88), showing comparable levels of general stress levels and burnout between nurses and physicians. However, that review found that overall mental health outcomes were lower for nurses, with higher levels of depression, posttraumatic stress disorder (PTSD), and anxiety. By virtue of the nature of their job, nurses are more psychologically and physically involved in patient care and for longer hours than physicians are, which could explain these results (88). Some studies in Belgium explored the working conditions of nurses, where they suffer from insufficient teamwork, organizational support, and social recognition (87, 89, 90). On the other hand, it is important to remember that there are several confounding factors to consider, such as gender proportions and mean age (88). Though there have been conflicting results regarding gender susceptibility to burnout in nurses, it has been reported that female nurses displayed higher levels of emotional exhaustion than male nurses (91). Burnout also presents higher incidences amongst young professionals under the age of thirty, possibly due to lack of experience and self-confidence exerting additional stress on their workload (92). The comparable burnout results between nurses and physicians may be understood best by the unique demands of the COVID-19 pandemic on these professionals. The increased workload, medical demand, and overall fear of COVID-19 due to proximity to patients were experienced by both physicians and nurses, explaining comparable burnout results (93).

Aside from understanding the trends of burnout in HCW, it is also important to address the results of this study and put forth solutions to this crisis. Aryankhesal et al. conducted a systematic review on effective interventions for burnout in nurses and physicians. Per their findings, psychosocial training and mindfulness techniques had a positive effect on improving mental health and burnout in nurses, whereas, e-mental health approaches, communication skills training, and online programs had a positive effect for physicians (94). Innovative approaches to encouragement and motivation were also shown to reduce burnout and improve mental health in nurses (95). For HCW in general, a frequent suggestion has been to increase the availability and accessibility of Personal Protective Equipment (PPE), as well training on their usage (96–98).

Other possible solutions to address burnout in HCW include recruitment and training of volunteers to relieve the heavy workload first and second-line workers experience (99). Longitudinal departmental burnout assessment as well as off-duty social gatherings which provide an opportunity to share challenges and boost morale are potential ways to keep track of workers mental health and assess stressors (10). Updating electronic medical record keeping techniques increases efficiency and minimizes the stress of documentation (100, 101). Lastly, providing stress management and resiliency training aid in addressing perceived loss of control and autonomy could prove helpful (10). The implementation of these interventions coupled with the understanding of burnout trends during the COVID-19 pandemic are useful steps in alleviating the healthcare crisis posed by burnout in HCW.

## 5. Limitations

High heterogeneity was found in the meta-analysis results; however, this was expected since the studies had been conducted in different periods of the pandemic and different countries. The studies included all had different sample sizes, ranging from 40 to 3,203, which also posed a limitation and contributed to the heterogeneity of the meta-analysis results. Additionally, the study outcomes were assessed using self-reported questionnaires in uncontrolled settings, therefore introducing reporting bias. The MBI used to assess burnout in most of the studies was not used in a standardized manner thus leading to differences in results; different Likert scales were used and cut off values for outcome results as well as definition of burnout varied across studies. To address the issue of different Likert scales, meta-analysis was conducted separately between studies that reported mean overall burnout and subscale score results using the same MBI version and Likert scale measure. To resolve the challenge proposed by inconsistent cut-off scores, the cut-off values used in this study were derived from a systematic review of 41 studies that used the MBI questionnaire for burnout evaluation (62). Included studies used different scales for burnout assessment which led to the exclusion of several studies from the meta-analysis. As sampling error is an inherent limitation in meta-analyses, it was not accounted for. Instead, studies were assessed using the Newcastle Ottawa Assessment Scale, which investigates sample selection and representativeness. Furthermore, handsearching of journals and recurrent searches on google scholar during the completion of the study in order to pick up new articles that might not be indexed in the databases selected was not performed, in accordance to Cochrane guidelines (37). At the time of this review, previous study had compared between early and late pandemic burnout or between region burnout, therefore limiting the discussion on the results of said subgroup analyses.

## 6. Conclusion

The purpose of this study was to explore the burnout prevalence in frontline medical healthcare and second-line workers during the COVID-19 pandemic as well as differences in prevalence according to region, healthcare profession and COVID-19 timeline. Our findings showed that frontline workers were at higher risk of experiencing burnout compared to their second-line workers colleagues, the early pandemic was associated with a higher burnout prevalence compared to late pandemic period and MENA had a higher burnout prevalence than Europe or South America. Burnout prevalence between physicians and nurses were found to be comparable. Though there have been studies on this phenomenon, this study specifically studied burnout in medical healthcare providers that work in a hospital setting. Possible solutions for burnout were also discussed, especially since burnout had significant consequential effects on HCW, patients, and the healthcare system. Further studies comparing burnout according to pandemic waves as well as regional analyses should be conducted so that more concrete evidence can be obtained on timeline and regional effects to better prepare for future pandemics A standardized diagnostic inventory for burnout assessment, as well as uniform cut-off scores, should be implemented to make measuring and grading of burnout easier.

## Data availability statement

The original contributions presented in this study are included in the article/Supplementary material, further inquiries can be directed to the corresponding author.

## Author contributions

MM: conceptualization, methodology, investigation, data curation, writing – original draft, writing – review and editing, visualization, and project administration. OS-O: investigation, data curation, writing – original draft, writing – review and editing, and visualization. RM: conceptualization, methodology, software, formal analysis, investigation, writing – review and editing, visualization, and supervision. AB: data curation, validation, and writing – review and editing. HN: conceptualization, validation, writing – review and editing, software, and supervision. CT and OA: conceptualization, project administration, writing – review and editing, and supervision. All authors contributed to the article and approved the submitted version.

## Supplementary material

Supplementary Item 1 Search strategy.

Supplementary Item 2 Methodological quality assessment.

Supplementary Item 3 Preferred reporting items for systematic reviews and meta-analyses (PRISMA) flow diagram.

Supplementary Item 4 Burnout scales.
