# Supplementary material for: A systematic review and meta analysis on burnout in physicians during the COVID-19 pandemic: A hidden healthcare crisis
Source: Front Psychiatry. 2023 Jan 12;13:1071397. doi: 10.3389/fpsyt.2022.1071397 (PMC9877514; doi:10.3389/fpsyt.2022.1071397)
Supplement: Supplementary Item 1 — Search strategy. [file Table_1.docx]

**Appendix 1. Actual Search Strategies**

**OVID**

Database(s): **Ovid MEDLINE(R) 1946 to Present and Epub Ahead of Print, In-Process & Other Non-Indexed Citations and Ovid MEDLINE(R) Daily, APA PsycInfo**1987 to November Week 1 2022**, EBM Reviews - Cochrane Central Register of Controlled Trials**October 2022**, EBM Reviews - Cochrane Database of Systematic Reviews**2005 to November 9, 2022**, Embase**1974 to 2022 November 11
Search Strategy:

| **#** | **Searches** |
| --- | --- |
| 1 | ((("Corona virinae" or "corona virus" or Coronavirinae or coronavirus or COVID or nCoV or hCoV) adj4 ("19" or "2019" or novel or new or nouveau or nuevo)) or (pneumon* adj3 Wuhan) or (("Corona virinae" or "corona virus" or Coronavirinae or coronavirus* or COVID or nCoV or hCoV) and (wuhan or china or chinese or hubei)) or "2019-novel Cov" or "2019-ncov" or "COVID-19" or "COVID2019" or "COVID 2019" or "Corona virinae19" or "Corona virinae2019" or "corona virus19" or "coronavirus-19" or "corona virus2019" or Coronavirinae19 or Coronavirinae2019 or coronavirus19 or coronavirus2019 or "coronavirus-2019" or COVID19 or COVID2019 or nCOV19 or ncov-19 or nCOV2019 or "SARS Corona virus 2" or "SARS-coronavirus2" or "SARS-coronavirus-2" or "SARS-COV-2" or "SARS-COV2" or "Severe Acute Respiratory Syndrome Corona virus 2" or "Severe Acute Respiratory Syndrome Coronavirus 2" or sarscov*).ti,ab,hw,kw,mp. or ((coronavir* or COVID*) adj5 (pandemic* or outbreak or shutdown or "shut down*" or "shut-down*" or quarantin* or (lock* adj down) or "lock-down*" or lockdown* or "stay at home" or "stay-at-home" or "shelter-in-place")).ti. or (Severe Acute Respiratory Syndrome Coronavirus 2 or COVID-19 or COVID-19 drug treatment or COVID-19 serotherapy or COVID-19 diagnostic testing or COVID-19 vaccine or spike glycoprotein, COVID-19 virus).os,ps,rs,ox,px,rx,nm. |
| 2 | (physician* or doctor* or interns or residency or "healthcare worker*" or "healthcare staff*").ti,ab,hw,kw. or *students, medical/ or exp medical staff/ or resident/ or ((house or attending) adj1 (physician* or staff or doctor*)).ti,ab,hw,kw. or faculty, medical/ |
| 3 | anesthesiology/ or clinical medicine/ or community medicine/ or emergency medicine/ or general practice/ or hospital medicine/ or internal medicine/ or military medicine/ or neurology/ or pediatrics/ |
| 4 | Physicians, Primary Care/ or exp Physicians/ or Physicians, Women/ or Physicians, Family/ |
| 5 | or/2-4 |
| 6 | ((burn* adj out) or burnout* or ((emotional* or psychological* or work or job) adj1 (stress* or distress*))).ti,ab,hw,kw. or burnout, professional/ or stress, psychological/ |
| 7 | (empath* or resilienc* or resilient or depression or "quality of life" or counsel* or "work-life" or depersonali* or workload or "sick leave" or absentee* or "emotional exhaustion" or "physical exhaust*" or anxiety or "well being" or "well-being" or wellbeing or suicid* or wellness).ti,ab,hw,kw. |
| 8 | exp psychotherapy/ or anxiety disorders/ or depressive disorders/ or depression/ or quality of life/ or occupational diseases/ or quality of life/ or occupational stress/ or "quality of work life"/ or work related illnesses/ or mental health/ |
| 9 | or/6-8 |
| 10 | 5 and 9 |
| 11 | anesthesiology/px, th or clinical medicine/px, th or community medicine/px, th or emergency medicine/px, th or general practice/px, th or hospital medicine/px, th or internal medicine/px, th or military medicine/px, th or neurology/px, th or pediatrics/px, th or Physicians, Primary Care/px, th or Physicians/px, th or Physicians, Women/px, th or Physicians, Family/ed, px, th or exp medical education/px, th |
| 12 | 10 or 11 |
| 13 | 1 and 12 |
| 14 | limit 13 to yr="2019 -Current" |
| 15 | remove duplicates from 14 |
| 16 | limit 15 to english language [Limit not valid in CDSR; records were retained] |
| 17 | limit 15 to no language specified [Limit not valid in CDSR; records were retained] |
| 18 | 16 or 17 |

**SCOPUS**

| 1 | ( TITLE-ABS-KEY ( ( ( "Corona virinae" OR "corona virus" OR coronavirinae OR coronavirus OR covid OR ncov OR hcov ) W/4 ( "19" OR "2019" OR novel OR new OR nouveau OR nuevo ) ) OR ( pneumon* W/3 wuhan ) OR ( ( "Corona virinae" OR "corona virus" OR coronavirinae OR coronavirus* OR covid OR ncov OR hcov ) AND ( wuhan OR china OR chinese OR hubei ) ) OR "2019-novel Cov" OR "2019-ncov" OR "COVID-19" OR "COVID2019" OR "COVID 2019" OR "Corona virinae19" OR "Corona virinae2019" OR "corona virus19" OR "coronavirus-19" OR "corona virus2019" OR coronavirinae19 OR coronavirinae2019 OR coronavirus19 OR coronavirus2019 OR "coronavirus-2019" OR covid19 OR covid2019 OR ncov19 OR ncov-19 OR ncov2019 OR "SARS Corona virus 2" OR "SARS-coronavirus2" OR "SARS-coronavirus-2" OR "SARS-COV-2" OR "SARS-COV2" OR "Severe Acute Respiratory Syndrome Corona virus 2" OR "Severe Acute Respiratory Syndrome Coronavirus 2" OR sarscov* ) ) OR ( TITLE ( ( coronavir* OR covid* ) W/5 ( pandemic* OR outbreak OR shutdown OR "shut down*" OR "shut-down*" OR quarantin* OR ( lock* w/ down ) OR "lock-down*" OR lockdown* OR "stay at home" OR "stay-at-home" OR "shelter-in-place" ) ) ) |
| --- | --- |
| 2 | (physician* or doctor* or interns or residency or "healthcare worker*" or "healthcare staff*") or ((house or attending) w/1 (physician* or staff or doctor*)) |
| 3 | ((burn* w/ out) or burnout* or ((emotional* or psychological* or work or job) w/1 (stress* or distress*))) |
| 4 | (empath* or resilienc* or resilient or depression or "quality of life" or counsel* or "work-life" or depersonali* or workload or "sick leave" or absentee* or "emotional exhaustion" or "physical exhaust*" or anxiety or "well being" or "well-being" or wellbeing or suicid* or wellness) |
| 5 | 3 or 4 |
| 6 | 1 and 2 and 5 |
| 7 | INDEX(embase) OR INDEX(medline) OR PMID(0* OR 1* OR 2* OR 3* OR 4* OR 5* OR 6* OR 7* OR 8* OR 9*) |
| 8 | 6 not 7 |
| 9 | DOCTYPE(ed) OR DOCTYPE(bk) OR DOCTYPE(er) OR DOCTYPE(no) OR DOCTYPE(sh) OR DOCTYPE(ch) |
| 10 | 9 not 10 |
| 11 | PUBYEAR AFT 2018 AND LANGUAGE(english) |
| 12 | 10 and 11 |
