# Supplementary material for: A systematic review and meta analysis on burnout in physicians during the COVID-19 pandemic: A hidden healthcare crisis
Source: Front Psychiatry. 2023 Jan 12;13:1071397. doi: 10.3389/fpsyt.2022.1071397 (PMC9877514; doi:10.3389/fpsyt.2022.1071397)
Supplement: Supplementary Item 2 — Methodological quality assessment. [file Table_2.docx]

Supplementary Items

| Author, Year | Question 1 | Question 2 | Question 3 | Question 4 | Question 5 | Question 6 | Question 7 | Total | Quality |
| --- | --- | --- | --- | --- | --- | --- | --- | --- | --- |
| Akova I, Kiliç E, & Ozdemir ME | * | * | NA | ** | NA | * | * | 6 stars |  |
| Alsulimani LK et al. | * | * | * | ** | NA | * | * | 6 stars |  |
| Alwashmi AH & Alkhamees AA | * | NA | NA | ** | NA | * | * | 5 stars |  |
| Appiani FJ. et al. | NA | NA | * | ** | NA | * | * | 5 stars |  |
| Asghar MS et al. | * | * | NA | ** | NA | * | * | 6 stars |  |
| Azoulay E et al. | * | NA | * | ** | NA | * | * | 6 stars |  |
| Babamiri M et al. | * | NA | * | ** | NA | * | * | 6 stars |  |
| Di Mattei VE. et al. | * | NA | NA | ** | NA | * | * | 5 stars |  |
| Dobson H et al. | * | NA | NA | ** | NA | * | * | 5 stars |  |
| Enea V et al. | * | NA | NA | ** | NA | * | * | 5 stars |  |
| Etesam F et al | * | NA | NA | ** | NA | * | * | 5 stars |  |
| Fumis RRL et al. | * | NA | * | ** | NA | * | * | 6 stars |  |
| Gupta MD et al | * | NA | NA | ** | NA | * | NA | 4 stars |  |
| Haji Seyed Javadi SA et al. | NA | NA | NA | ** | NA | * | * | 4 stars |  |
| Ibar C et al. | NA | NA | NA | ** | NA | * | * | 4 stars |  |
| Ismail TI et al. | * | NA | NA | ** | NA | * | * | 5 stars |  |
| Jiang W et al. | * | NA | NA | ** | NA | * | * | 5 stars |  |
| Kanneganti A et al. | * | NA | NA | ** | NA | * | * | 5 stars |  |
| Kapetanos et al | * | NA | NA | ** | NA | * | * | 5 stars |  |
| Karacan FA et al. | * | * | NA | ** | NA | * | * | 6 stars |  |
| Kashtanov A et al. | * | NA | NA | ** | NA | * | * | 5 stars |  |
| Khan N et al. | NA | NA | NA | ** | NA | * | * | 4 stars |  |
| Khoudoruth MAS et al. | * | NA | NA | ** | NA | * | * | 5 stars |  |
| Kim C et al. | * | NA | NA | ** | NA | * | * | 5 stars |  |
| Mendonça VS, Steil A, & Teixeira de Gois AF | * | NA | NA | ** | NA | * | * | 5 stars |  |
| Mosolova E, Sosin D, & Mosolov S | * | NA | NA | ** | NA | * | * | 5 stars |  |
| Mousavi-Asl B et al. | NA | NA | NA | ** | NA | * | * | 4 stars |  |
| Mutleq A et al. | * | NA | NA | ** | NA | * | NA | 4 stars |  |
| Naldi A et al. | * | * | NA | ** | NA | * | * | 6 stars |  |
| Queiroz de Paiva Faria AR et al. | * | NA | NA | ** | NA | * | * | 5 stars |  |
| Ruiz-Fernandez MD et al. | * | NA | NA | ** | NA | * | * | 5 stars |  |
| Sarikhani Y et al. | * | NA | NA | ** | NA | * | * | 5 stars |  |
| Shiu C et al. | * | NA | NA | ** | NA | * | * | 5 stars |  |
| Singh et al. | * | NA | NA | ** | NA | * | * | 5 stars |  |
| Steil A. et al. | * | NA | NA | ** | NA | * | * | 5 stars |  |
| Stocchetti N et al. | * | NA | NA | ** | NA | * | * | 5 stars |  |
| Teo I et al. | * | NA | NA | ** | NA | * | * | 5 stars |  |
| Torrente M et al. | * | NA | NA | ** | NA | * | * | 5 stars |  |
| Treluyer L & Tourneaux P | * | * | NA | ** | NA | * | * | 6 stars |  |
| Tuna T. & Ozdin S | * | NA | NA | ** | NA | * | * | 5 stars |  |
| Turan C. et al. | NA | NA | NA | ** | NA | * | * | 4 stars |  |
| Yilmaz Y, Erdogan A, & Bahadir E. | * | NA | NA | ** | NA | * | * | 5 stars |  |
| Zakaria MI et al. | NA | NA | * | ** | NA | * | * | 6 stars |  |
| Zhang X et al. | * | * | * | ** | NA | * | * | 7 stars |  |
| Zhou et al | * | NA | NA | ** | NA | * | * | 5 stars |  |

Abbreviations:

*NA: Not Applicable*

**Supplementary Item 2:** Methodological Quality Assessment

Selection (Maximum 5 stars)

Question 1: Was the sample representative of the target population?

a) Truly representative of the average in the target population. * (all subjects or random sampling)

b) Somewhat representative of the average in the target population. * (non-random sampling)

c) Selected group of users.

d) No description of the sampling strategy.

Question 2: Was the sample size justified and satisfactory?

a) Justified and satisfactory. *

b) Not justified.

Question 3: Was the comparability between respondents and non-respondents characteristics established, and was the response rate satisfactory?

a) Comparability between respondents and non-respondents characteristics is established, and the response rate is satisfactory. *

b) The response rate is unsatisfactory, or the comparability between respondents and non-respondents is unsatisfactory.

c) No description of the response rate or the characteristics of the responders and the non-responders.

Question 4: How was the outcome ascertained?

a) Validated measurement tool. **

b) Non-validated measurement tool, but the tool is available or described.*

c) No description of the measurement tool.

Comparability (Maximum 2 stars)

Question 5: Were the subjects in different outcome groups comparable, based on the study design or analysis?

a) The study controls for the most important factor (select one). *

b) The study control for any additional factor. *

Outcome (Maximum 3 stars)

Question 6: How was the outcome measured?

a) Independent blind assessment. **

b) Record linkage. **

c) Self report. *

d) No description.

Question 7: Was the statistical test used clearly described and appropriate?

a) The statistical test used to analyze the data is clearly described and appropriate, and the measurement of the association is presented, including confidence intervals and the probability level (p value). *

b) The statistical test is not appropriate, not described or incomplete.

Thresholds for converting the Newcastle-Ottawa scales to AHRQ standards (good, fair, and

poor):

- Good quality: 3 or 4 stars in selection domain AND 1 or 2 stars in comparability domain AND 2 or 3 stars in outcome/exposure domain
- Fair quality: 2 stars in selection domain AND 1 or 2 stars in comparability domain AND 2 or 3 stars in outcome/exposure domain
- Poor quality: 0 or 1 star in selection domain OR 0 stars in comparability domain OR 0 or 1 stars in outcome/exposure domain
