# Supplementary material for: A systematic review and meta analysis on burnout in physicians during the COVID-19 pandemic: A hidden healthcare crisis
Source: Front Psychiatry. 2023 Jan 12;13:1071397. doi: 10.3389/fpsyt.2022.1071397 (PMC9877514; doi:10.3389/fpsyt.2022.1071397)
Supplement: Supplementary Item 3 — Preferred reporting items for systematic reviews and meta-analyses (PRISMA) flow diagram. [file Table_3.docx]

Supplementary Items

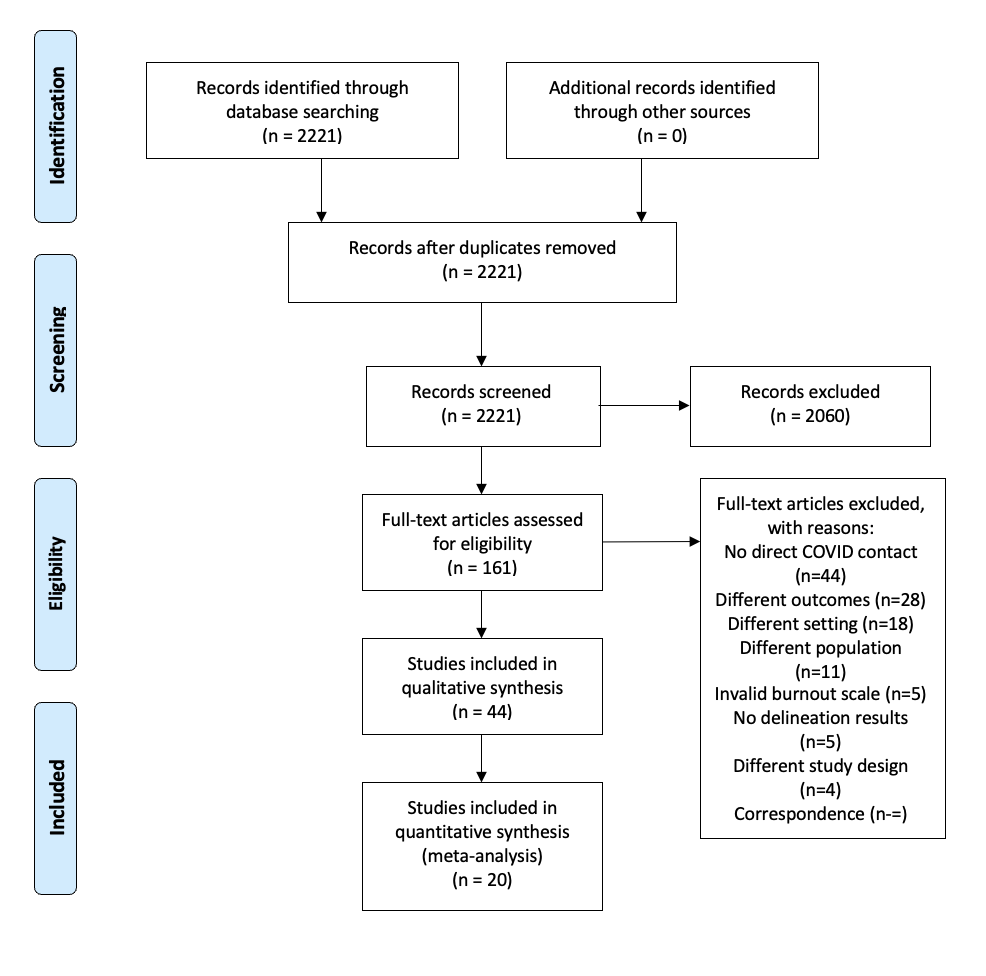


**Supplementary item 3**: Preferred Reporting Items for Systematic Reviews and Meta-Analyses (PRISMA) Flow Diagram
