# Supplementary material for: A systematic review and meta analysis on burnout in physicians during the COVID-19 pandemic: A hidden healthcare crisis
Source: Front Psychiatry. 2023 Jan 12;13:1071397. doi: 10.3389/fpsyt.2022.1071397 (PMC9877514; doi:10.3389/fpsyt.2022.1071397)
Supplement: Supplementary Item 4 — Burnout scales. [file Table_4.docx]

Supplementary Items

| Citation | MBI | ProQOL | OLBI | CBI | PFI | Mini-Z | Others |
| --- | --- | --- | --- | --- | --- | --- | --- |
| Akova I, Kiliç E, & Ozdemir ME | 5 point Likert .  For EE  ≤20 low, 21–27 moderate, and ≥28 high.  For DP  ≤8 were evaluated as low, 9–12 as moderate, and ≥13 as high.  For PA  ≤23 were evaluated as low, 24–27 as moderate, and ≥28 as high. | - | - | - | - | - | - |
| Alsulimani LK et al. | - | - | - | No detail given on Likert scale used  Used the work-related burnout part only. Consists of 7 questions, each question answer was given a score ranging from 0.0, 0.25, 0.50, 0.75 to 1.0. If less than 4 questions were answered, the respondent was classified as a non-responder. A final score of 2.99 or lower indicated no job burnout, while a score of 3.00 or higher was interpreted as indicating job burnout. | - | - | - |
| Alwashmi AH & Alkhamees AA | 7 point Likert  Scoring 27 or more on EE, 13 or more on DP or 31 or less on PA was highly indicative of burnout. | - | - | - | - | - | - |
| Appiani FJ. et al. | 7 point Likert  A score of ≥26 for EE, ≥9 for DP and/or ≤36 for PA was defined as the presence of burnout syndrome. | - | - | - | - | - | - |
| Asghar MS et al. | 7 point Likert  The cutoff set for EE was high if > 27, moderate when 17- 26, and low if < 17.  DP was also set as high > 13, moderate 7–12, and low <7.  PA was  high if > 39, moderate when 32-38,and low if < 32. | - | - | - | - | - | - |
| Azoulay E et al. | 7 point Likert  severe burnout was defined as an MBI score > 9. | - | - | - | - | - | - |
| Babamiri M et al. | 7 point Likert  cutoffs for moderate and severe EE being ≥ 17 and ≥ 27,  moderate and severe DP being ≥ 7 and ≥ 13 respectively, and moderate to severe reduced PA being ≤ 38 and ≤ 21 respectively. | - | - | - | - | - | - |
| Di Mattei VE. et al. | 7 point Likert  EE subscale scores were divided as low (≤ 14), medium (15–23), and high (≥ 24); the  DP subscale scores were grouped into low (≤ 3), medium (4-8), and high ((≥ 9);  and the reduced PA subscale scores are classified into low ( ≥ 37), medium  (30-36), and high (≤ 29). | - | - | - | - | - | - |
| Dobson H et al. | - | - | - | - | 5 point Likert scale  No details given on cut-off scores. | - | - |
| Enea V et al. | - | - | - | 5 point Likert  No details given on cut-off scores. | - | - | - |
| Etesam F et al | 7 point Likert  No details given on cut-off scores. | - | - | - | - | - | - |
| Fumis RRL et al. | 5 point Likert  Definition of burnout was adopted,  according to the MBI manual: high levels of EE (score ≥27 points) and DP (score ≥10 points) combined with low PA (score ≤33 points). | - | - | - | - | - | - |
| Gupta MD et al | - | - | - | - | - | No details given on Likert scale used  Burnout was indicated by a score ≥ 3 on Mini-Z burnout item. | - |
| Haji Seyed Javadi SA et al. | 7 point Likert  No details given on cut-off scores. | - | - | - | - | - | - |
| Ibar C et al. | 7 point Likert  the definition of burnout requires the presence of the three  subscales: high EE (score > 26), high DP (score > 9) and low PA (score <  34). | - | - | - | - | - |  |
| Ismail TI et al. | 7 point Likert  The level of burnout (EE score) can vary between 9 and 63, a score of 9 ± 18 signifying a low level, 18 ± 29 a moderate level, and values higher than 29 depicting severe burnout. | - | - | - | - | - | - |
| Jiang W et al. | - | 5 point Likert  A raw score of ≤ 22 indicated a low level of burnout. | - | - | - | - | - |
| Kanneganti A et al. | - | - | 4 point Likert scale  A score of ≥2.25 for Exhaustion or ≥2.10 for Disengagement correlated with physical symptoms and was used to as a cut-off to define burnout. | - | - | - | - |
| Kapetanos et al | 7 point Likert scale  cut-off of more of equal to 27 indicated high EE, more or equal to 10 indicated high DP,  and <34 indicated low PA. | - | - | - | - | - | - |
| Karacan FA et al. | 7 point Likert scale  No detail was given regarding cut-offs. | - | - | - | - | - | - |
| Kashtanov A et al. | 7 point Likert scale  EE was divided into low level (0-15), average level (16-24) and high level (≥ 25)  - DP was divided into low level (0-5), average (5-10), high level (≥ 11)  - PA was divided into low level (≥ 37) average level (31-36), and high level is (0-30). | - | - | - | - | - | - |
| Khan N et al. | 7 point Likert  The presence of physician burnout was defined as an EE score ≥27 or DP score ≥10. Feeling low PA (defined as a score ≤33) was evaluated separately from overall burnout. | - | - | - | - | - | - |
| Khoudoruth MAS et al. | - | No details given on Likert scale used  Higher score (>41) indicated higher risk for burnout). | - | - | - | - | - |
| Kim C et al. | - | 5 point Likert scale  No details given on cut-off scores. | - | - | - | - | - |
| Mendonça VS, Steil A, & Teixeira de Gois AF | - | - | No details given on Likert scale used  Burnout was defined as positive if the total score on OLBI was 21. | - | - | - | - |
| Mosolova E, Sosin D, & Mosolov S | 7 point Likert  Low EE (0-1), moderate EE (2-3), high EE (4-6). Cut-off for low, moderate and high DP was same. | - | - | - | - | - | - |
| Mousavi-Asl B et al. | 7 point Likert  A high score in EE and DP or a low score in PA showed a high level of occupational burnout. Scores ≥ 37 for EE, ≥ 13 for DP, and ≥ 39 for PA were assumed high. | - | - | - | - | - | - |
| Mutleq A et al. | - | - | 5 point Likert scale  In order to limit the study to burnout related to COVID-19, the phrase “caused by COVID-19” was added to each item.  Cut-off value for total burnout score was 2.25. | - | - | - | - |
| Naldi A et al. | 7 point Likert  EE scores were 15–23, PA scores were 30–36 and DP scores were 4–8; and low if EE scores were ≤14, PA scores were ≥37 and DP scores were ≤3. | - | - | - | - | - | - |
| Queiroz de Paiva Faria AR et al. | 5 point Likert  Individuals with mean response options equal to or higher than “sometimes” (a score of 3 on the Likert scale) for the EE and DP dimensions or scores of 3 or less for the rPA dimension were considered to have a high risk of BS. | - | - | - | - | - | - |
| Ruiz-Fernandez MD et al. | - | This is a self-report questionnaire consisting of 30 items rated on a 6-point Likert scale (ranging from 0 = “never” to 5 = “always”). The scale is divided into three subscales: Compassion fatigue (10 items), Compassion satisfaction (10 items) and Burnout(10 items). Higher scores in each of the dimensions indicate higher levels of CF, CS and BO, respectively. Scores can be categorised as low, medium and high in each of the three subscales: CF (≤8, low; 9–17, medium; and ≥17, high), CS (≤33, low; 34–41, medium; and ≥42, high) and BO (≤18, low; 19–26, me-dium; and ≥27 high). | - | - | - | - | - |
| Sarikhani Y et al. | 7 point Likert  EE was classified high if (> 26), intermediate  (17-26), and low if (< 17). For DP, high if (> 12), intermediate (7-12) and low if (< 7). For PA, high if (> 39), moderate (32-38), and low (< 32). An EE score equal to or  more than 27 or a DP score equal to or more than 13 was  considered overall burnout. | - | - | - | - | - | - |
| Shiu C et al. | - |  |  |  |  |  | 5 point Likert scale  Burnout was assessed using a single item developed with 5404 primary care providers and results showed this assessment had strong psychometric validity and good sensitivity (83.2%) and specificity (87.4%). The question asked “Overall, based on your definition of burnout, how would you rate your level of burnout?” The responses were dichotomized into 0 (did not burnout, including 1 and 2) and 1 (Burned out, including 3, 4, and 5). |
| Singh et al. | Modified 7 point Likert (excluded option 2 on 0-6 range). Used only EE and DP subscales. Scores were categorized  using cutoffs (EE score ≥ 9, DP score ≥ 6) to indicate high EE and DP. | - | - | - | - | - | - |
| Steil A. et al. | - | - | No details given on Likert scale used  Burnout was defined as positive if the total score on OLBI was 21. | - | - | - | - |
| Stocchetti N et al. | 7 point Likert  Burnout was considered high if EE scores were ≥24, PA scores were ≤29, and DP scores were ≥9; moderate if EE scores were 15–23, PA scores were 30–36, and DP scores were 4–8; and low if EE scores were ≤14, PA scores were ≥37, and DP scores were ≤3. | - | - | - | - | - | - |
| Teo I et al. | - | - | - | - | - | - | One-item burnout question from the Physician Work Life Scale where a score ≥ 3 indicating symptoms of burnout. |
| Torrente M et al. | 5 point Likert  Burn-out syndrome was defined to have a high percentile of EE, and/or a high percentile of DP and/or a low percentile of personal achievement. The median (IQR) scores on the classification of burn-out syndrome were defined as high level of EE 26,2 (20–32) and/or high level of DP 11,6 (ranged 9–14) and low level of PA 29,6 (ranged 26–34). | - | - | - | - | - | - |
| Treluyer L & Tourneaux P | 8 point Likert  The cut-off scores for the EE and DP domains were respectively ≥ 30 and ≥ 12. The cut-off for reduced PA was ≤ 33. Residents were considered to have experienced burnout when the level of EE or DP exceeded the cut-off, regardless of the presence or absence of reduced PA. | - | - | - | - | - | - |
| Tuna T. & Ozdin S | 5 point Likert  No details given on cut-off scores. | - | - | - | - | - | - |
| Turan C. et al. | 5 point Likert  No details given on cut-off scores. | - | - | - | - | - | - |
| Yilmaz Y, Erdogan A, & Bahadir E. | 5 point Likert  Mentioned that higher scores indicated burnout but did not specify cut-off scores. | - | - | - | - | - | - |
| Zakaria MI et al. | - | - | - | - | - | - | The questionnaire form was adopted from Michelle Post, Public Welfare, Vol. 39, No. 1, 1981, American Public Welfare Association.  5 point Likert scale  Scores 28–38 indicated burnout proof. 39–50 indicated unlikely burnout.  51–70 indicated a fair chance of burnout.  71–90 indicated early burnout. ≥ 91 indicated advance burnout. |
| Zhang X et al. | 7 point Likert  Burnout was defined as high EE (scores ≥ 27) and/or high DP (scores ≥ 10) as opposed to a total score.  -Higher scores on the EE and DP subscales indicated a higher burnout symptom, while PA was inversely associated with burnout. The low PA defined as scores ≤ 33. HCWs were categorised as having a high level of burnout if they scored high on EE and DP and low on PA. | - | - | - | - | - | - |
| Zhou et al | 7 point Likert  The degree of burnout was determined to be high when the scores in exhaustion and cynicism were high while the scores in professional efficacy is low. No details given on cut-off scores. | - | - | - | - | - | - |

*Abbreviations used*

*CBI= Copenhagen Burnout Inventory, EE= Emotional Exhaustion, DP= Depersonalization, MBI= Maslach Burnout Inventory, OLBI= Oldenburg Burnout Inventory, Mini Z= Zero Clinical Questionnaire, PFI= Professional Fulfillment Index, ProQOL= Professional Quality of Life.*

**Supplementary Item 4:** Burnout definition and cut-off scores according to each included study
